# A novel HSP90 inhibitor targeting the C-terminal domain attenuates trastuzumab resistance in HER2-positive breast cancer

**DOI:** 10.1186/s12943-020-01283-6

**Published:** 2020-11-20

**Authors:** Jung Min Park, Yoon-Jae Kim, Soeun Park, Minsu Park, Lee Farrand, Cong-Truong Nguyen, Jihyae Ann, Gibeom Nam, Hyun-Ju Park, Jeewoo Lee, Ji Young Kim, Jae Hong Seo

**Affiliations:** 1 Division of Medical Oncology, Department of Internal Medicine, Korea University College of Medicine, Korea University, Seoul, 152-703 Republic of Korea; 2 Brain Korea 21 Program for Biomedical Science, Korea University College of Medicine, Korea University, Seoul, 152-703 Republic of Korea; 3 Department of Biomedical Research Center, Korea University Guro Hospital, Korea University, Guro Hospital Campus, 97 Gurodong-gil, Guro-gu, Seoul, 08308 Republic of Korea; 4 Adelaide Medical School, Faculty of Health and Medical Sciences, The University of Adelaide, Adelaide, South Australia 5000 Australia; 5 Laboratory of Medicinal Chemistry, College of Pharmacy, Seoul National University, Seoul, 08826 Republic of Korea; 6 School of Pharmacy, Sungkyunkwan University, Suwon, Gyeonggi-do 16419 Republic of Korea

**Keywords:** C-terminal HSP90 inhibitor, NCT-547, HER2-positive breast cancer, Cancer stem cells, Trastuzumab resistance, p95HER2, HER2

## Abstract

Trastuzumab resistance in HER2-positive breast cancer is associated with a poorer prognosis. HSP90 is thought to play a major role in such resistance, but N-terminal inhibitors of this target have had little success. We sought to investigate the utility of NCT-547, a novel, rationally-designed C-terminal HSP90 inhibitor in the context of overcoming trastuzumab resistance. NCT-547 treatment significantly induced apoptosis without triggering the heat shock response (HSR), accompanied by caspase-3/− 7 activation in both trastuzumab-sensitive and -resistant cells. NCT-547 effectively promoted the degradation of full-length HER2 and truncated p95HER2, while also attenuating hetero-dimerization of HER2 family members. The impairment of cancer stem-like traits was observed with reductions in ALDH1 activity, the CD24^low^/CD44^high^ subpopulation, and mammosphere formation in vitro and in vivo. NCT-547 was an effective inhibitor of tumor growth and angiogenesis, and no toxic outcomes were found in initial hepatic and renal analysis. Our findings suggest that NCT-547 may have applications in addressing trastuzumab resistance in HER2-positive breast cancer.

**Supplementary Information:**

The online version contains supplementary material available at 10.1186/s12943-020-01283-6.

## Main text

HSP90 is an important protein chaperone that responds to stress conditions by maintaining the integrity of protein synthesis and folding for cellular homeostasis [1]. HER2 is one such potential oncogenic protein amongst the many HSP90 clients. HSP90 directly modulates HER2 kinase activity, which affects downstream signaling. Despite the improvements in clinical outcomes enabled by trastuzumab, most patients will eventually become resistant to the drug with recurrence of the disease and metastasis [1, 2].

Trastuzumab resistance has been correlated to both EGFR/HER2 and HER2/HER3 heterodimers generating aberrant compensatory signaling, rendering anti-HER2 therapy ineffective [2]. Another reported mechanism arises from the truncated form of HER2 (known as p95HER2) that shows steric effects leading to constitutive HER2 kinase activity. Oncogenic p95HER2 is also a HSP90 client protein and shows a reliance on the HSP90 chaperone complex [3]. These findings suggest that the inhibition of HSP90 in HER2-positive breast cancer could serve to overcome trastuzumab resistance and improve anti-tumor effects.

HSP90 inhibitors developed in recent years have primarily targeted the N-terminal domain of HSP90. However, no candidates have been approved to date, due to issues including poor solubility and organ impairment caused by off-target toxicity [4]. HSF-1 is a key effector in the HER2 signaling pathway and is responsible for a comprehensive range of pro-survival effects as well as chemoresistance. N-terminal inhibitors trigger HSF-1 activation, resulting in increased transcription of HSP family members including HSP27, HSP70 and HSP90. This event is collectively referred to as the heat shock response (HSR) and is a pro-survival pathway for malignant cells [5]. In this context, C-terminal inhibition of HSP90 represents an alternative strategy that could ameliorate the current drawbacks of N-terminal HSP90 inhibitors [4].

## Results and discussion

### NCT-547 induces apoptosis and targets HER2 signaling

**Fig. 1:**
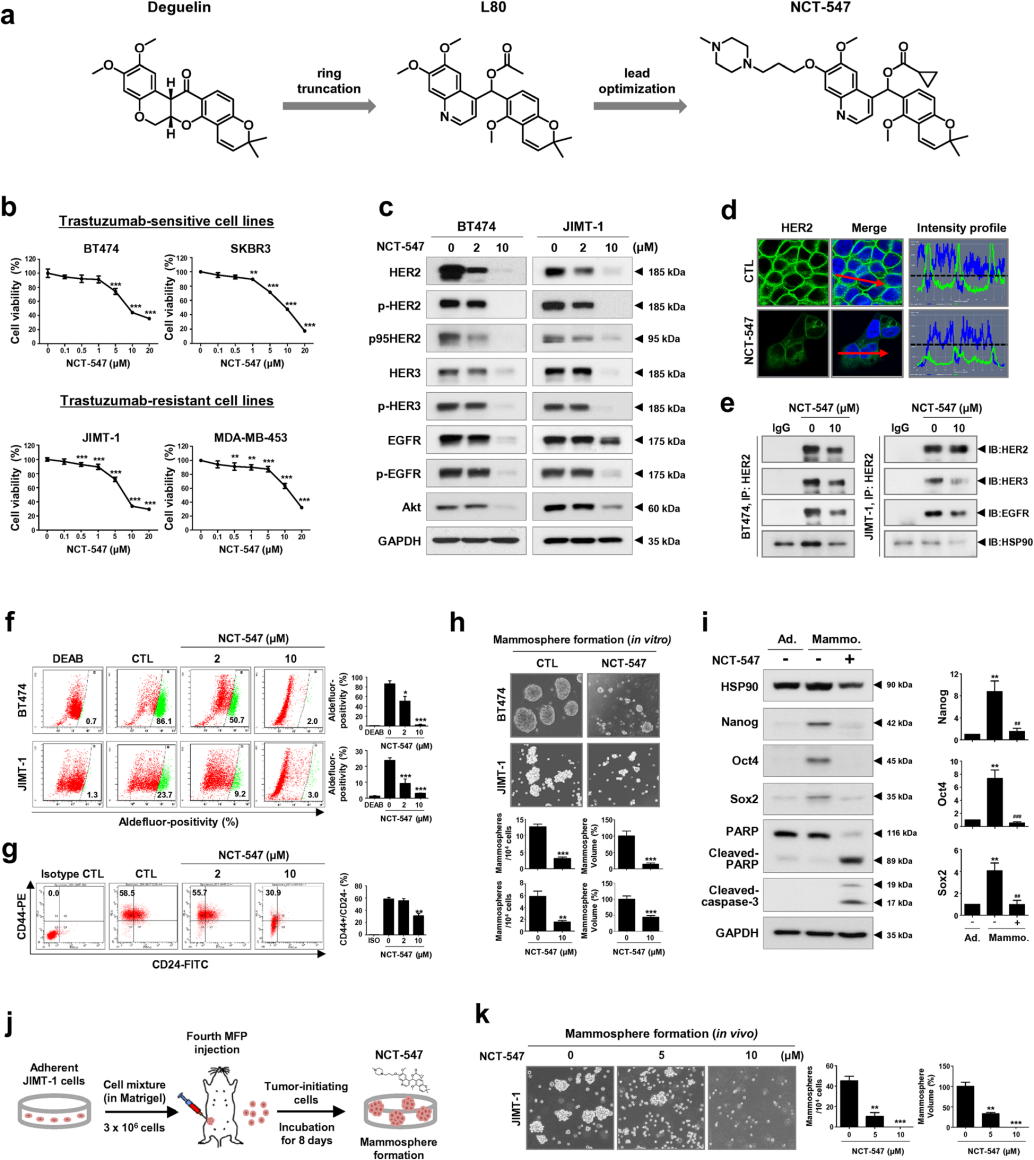
NCT-547 targets HER2 signaling and cancer stem-like properties in HER2-positive breast cancer cells. **a** Chemical structures of NCT-547. NCT-547 was synthesized as a lead-optimized product of L80, which is a C-ring truncated deguelin derivative. **b** HER2-positive cell lines (BT474, SKBR3, JIMT-1, and MDA-MB-453) were treated with the indicated concentrations of NCT-547 (0–20 μM) for 72 h. **c-e** NCT-547 inhibits HER2 signaling. **c** Reduced expression of full-length p185HER2, p95HER2, phospho-HER2 (Tyr1221/1222), HER3, phospho-HER3 (Tyr1289), EGFR, phospho-EGFR, and Akt observed following exposure to NCT-547 (0–10 μM, 72 h) by immunoblot analysis. **d** Immunocytochemical analysis and intensity profiling for HER2. BT474 cells were treated with NCT-547 (10 μM, 24 h) and immunostained for HER2 (1:100, green) with DAPI nuclear staining (blue). The intensity (y-axis, green) of HER2 signal in the plasma membrane is represented in arbitrary units as defined by the software. **e** BT474 and JIMT-1 cells were treated with NCT-547 (0–10 μM) for 24 h. Cells were immunoprecipitated with HER2 antibody and analyzed by immunoblotting of HER2, HER3, EGFR and HSP90 antibodies. IP, immunoprecipitation; IB, immunoblot; IgG, normal rabbit immunoglobulin G. **f-k** NCT-547 suppresses cancer stem-like properties. **f** Aldefluor-positivity in BT474 and JIMT-1 cells as measured by flow cytometry after exposure to NCT-547 (0–10 μM, 72 h) and Aldefluor-positive cells were quantified (*p < 0.05). **g** CD44+/CD24- population in the JIMT-1 cells was analyzed by flow cytometry after NCT-547 treatment (0–10 μM) for 72 h (**p < 0.01). **h** Effect of NCT-547 on mammosphere formation by BT474 and JIMT-1 cells was demonstrated by 3D-culturing cells in ultralow attachment plates in the presence or absence of NCT-547 (0–10 μM, 72 h). The numbers and sizes of BT474- and JIMT-1-mammospheres were significantly reduced after exposure to NCT-547 (**p < 0.01). **i** Effect of NCT-547 (10 μM, 72 h) on expression of stemness-related factors in BT474 mammospheres. Changes in HSP90, Nanog, Oct4 and Sox2, PARP and cleaved caspase-3 levels as determined by immunoblotting. Quantitative graphs of Nanog, Oct4 and Sox2 expression [**p < 0.01, adherent cells (Ad.) vs mammospheres (Mammo.); ##*p* < 0.01, DMSO control vs NCT-547 treatment in mammospheres]. **j-k** Effect of NCT-547 on mammosphere formation in a JIMT-1 xenograft model. Dissociated single cells (1 × 10^6^/ml) from primary tumors (200 ~ 250 mm^3^) were plated in ultralow attachment dishes and cultured in the presence or absence of NCT-547 (0–10 μM) for 8 days. The numbers and volumes of mammospheres were quantified (**p < 0.01)

We previously synthesized the C-ring truncated deguelin derivative L80 as a C-terminal HSP90 inhibitor and demonstrated that it elicits anti-metastatic activity in TNBC via suppression of STAT3 signaling [6]. NCT-547 is a lead-optimized product of L80 discovered through an investigation of the structure–activity relationship (Fig. 1a and Additional file 3: Figure S1). We first sought to evaluate the effect of NCT-547 on cell viability and apoptosis in HER2-positive breast cancer cell lines, including trastuzumab-sensitive BT474 and SKBR3, and trastuzumab-resistant JIMT-1 and MDA-MB-453 cells. Cell viability in both trastuzumab-sensitive and -resistant cells was dose-dependently reduced by NCT-547 (**p < 0.01, Fig. 1b and Additional file 3: Figure S2). NCT-547-induced apoptosis was observed in these cells, accompanied by increased sub-G1 accumulation and caspase-3/− 7 activation (Additional file 3: Figure S3). In contrast, NCT-547 had no significant effect on the non-malignant cell lines HEK293 and MCF10A (Additional file 3: Figure S4).

The expression levels and phosphorylation of HER2, HER3 and EGFR were significantly reduced, together with Akt downregulation in both BT474 and JIMT-1 cells following NCT-547 challenge (Fig. 1c and d, Additional file 3: Figure S5). NCT-547 also downregulates truncated-p95HER2, which has tyrosine kinase activity. Immunoprecipitation assays with anti-HER2 antibodies revealed that NCT-547 reduced the presence of HER2/HER3 and HER2/EGFR heterodimers, as well as blocked the interaction between HSP90 and HER2 in BT474 and JIMT-1 cells (Fig. 1e).

### NCT-547 targets BCSC-like properties

Cancer stem cells (CSCs) have been implicated in drug resistance and metastatic relapse. A positive correlation has been reported between overexpression of HSP90 and ALDH-positive BCSCs [7]. Impaired BCSC-like properties including ALDH1 activity, the CD44+/CD24- population and mammosphere-forming capacity were observed in response to NCT-547 (*p < 0.05, Fig. 1f-h). Consistent with in vitro observations, mammospheres with highly enriched BCSC-like populations in trastuzumab-resistant tumors in vivo were significantly inhibited (**p < 0.01, Fig. 1j and k). Of particular note, stemness factors such as Nanog and Oct4 are described as potential HSP90 client proteins [6, 8]. The elimination of BCSC-subpopulations was observed via decreased levels of Nanog, Oct4 and Sox2 as well as the caspase-3-mediated apoptotic pathway, implying that NCT-547 effectively kills both proliferating and cancer stem-like cells (**p < 0.01, ##*p* < 0.01, Fig. 1i).

### C-terminal HSP90 inhibitor NCT-547 does not induce the heat shock response (HSR)

**Fig. 2:**
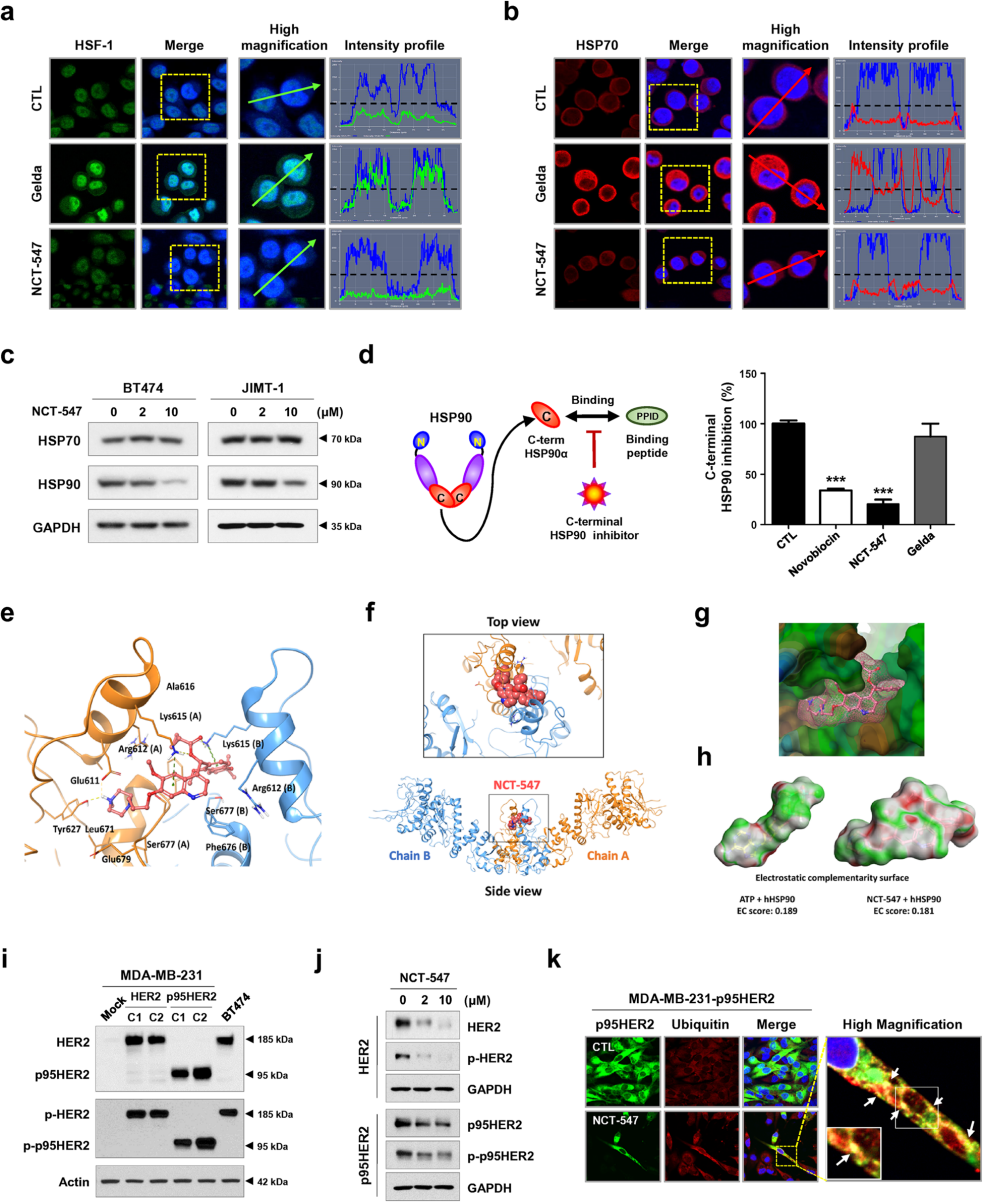
NCT-547 targets the C-terminal binding site of HSP90. **a-b** SKBR3 cells immunostained for HSF-1 (red, **a**) and HSP70 (green, **b**) with DAPI (blue) after exposure to NCT-547 (300 nM) and geldanamycin (300 nM) for 24 h. Intensity of nuclear HSF-1 (green) and cytosolic HSP70 (red) is represented in arbitrary units as defined by the software using the intensity profile tool. Gelda; geldanamycin. **c** No change in protein levels of HSP70 was observed, whereas HSP90 expression was reduced in the presence of NCT-547 (0–10 μM, 72 h). **d** HSP90α (C-terminal) Inhibitor Screening Assay was used to assess inhibition of the C-terminal binding domain of HSP90 by the inhibitors (novobiocin, gelda and NCT-547). The activity of HSP90 inhibitors was measured with an Alphascreen microplate reader, and the inhibitory effect of each drug was determined at 500 μM. **e-h** Structural modeling of docking between NCT-547 and hHSP90. **e** Binding pose of NCT-547 in the C-terminal domain of open state hHSP90 (Surflex-Dock score = 10.228, CScore = 2). Chain A of hHSP90 is rendered as an orange ribbon, and chain B is sky blue. NCT-547 as a ball-and-stick model. Hydrogen bonds and π-cation interactions are represented as yellow and green dashed lines respectively. **f** View of the entire binding pose of NCT-547 at the dimerization interface. NCT-547 as a space-filling model. **g** Lipophilicity property surface map (brown color: hydrophobic, blue color: hydrophilic) of the active site. Connolly surface of NCT-547 is shown as pink mesh. **h** Comparison of the electrostatic complementarity (EC) surface and EC score of ATP with those of NCT-547. Green = perfect electrostatic complementarity (1), grey = both potentials zero, red = perfect electrostatic clash (− 1). **i-k** NCT-547 degrades HER2 and p95HER2 in HER2- and p95HER2- overexpressing MDA-MB-231 cancer cells. **i** The expression of HER2, p95HER, phospho-HER2, and phospho-p95HER2 was upregulated in both HER2- and p95HER2-overexpressing MDA-MB-231 (two clones; C1 and C2) compared to the parental MDA-MB-231 cells. **j** Levels of full-length p185HER2, p95HER2, phospho-HER2 (Tyr1221/1222) and phospho-p95HER2 were detected after treatment of NCT-547 (0–10 μM, 72 h) by immunoblot analysis. **k** p95HER2-overexpressing MDA-MB-231 cells were co-immunostained for p95HER2 (1:100, green) and ubiquitin (1:100, red) with DAPI (blue) after exposure to NCT-547 (10 μM) for 24 h. Co-localization of ubiquitin and p95HER2 in the plasma membrane is seen as yellow signal (white arrows) at high magnification (× 2000)

Compelling evidence suggests that HSR induced by N-terminal targeting HSP90 inhibitors is a major obstacle that impedes anti-tumor activity [4]. HER2 promotes constitutive activation of the HSF1-HSP90 axis, accompanied by upregulation of HSP70 which is associated with reduced sensitivity to HSP90 inhibitors, thus circumventing the cellular induction of apoptosis [9]. To address this unmet need, dual therapeutic inhibition of HSP90/HSP70 may be desirable. We observed that C-terminal inhibition neither increased levels of the compensatory pro-survival factor HSP70 nor promoted the nuclear accumulation of HSF-1 in HER2-positive breast cancer cells (Fig. 2a-c, Additional file 3: Figure S6), suggesting that NCT-547 may have substantial advantages by targeting HSP90 without affecting HSF-1 transcriptional activity.

To confirm the interplay between NCT-547 and C-terminal binding site of HSP90, we conducted a specific HSP90α C-terminal inhibitor screening assay. Compared with geldanamycin, NCT-547 significantly restrained C-terminal HSP90 activity (***p < 0.001, Fig. 2d), with an effect that appeared to be superior to novobiocin, a potent C-terminal inhibitor. To investigate the possible binding mode of NCT-547 to the ATP-binding site of C-terminal domain hHSP90, molecular docking was performed. NCT-547 theoretically docks well with the C-terminal domain of the hHSP90 homodimer and stabilizes the open conformation. The quinoline and N-methylpiperazine moiety show electrostatically favorable interactions with hHSP90 (green), and the EC score of NCT-547 (0.181) is similar to ATP (0.189) although NCT-547 is much larger than ATP (Fig. 2e-h).

### NCT-547 degrades HER2 and p95HER2 in HER2- and p95HER2- overexpressing MDA-MB-231 cells

Stable HER2- and p95HER2-overexpressing cell lines were generated from MDA-MB-231 TNBC cells lacking HER2 expression to examine whether NCT-547 could effectively degrade HER2 and p95HER2. While HER2-overexpressing cells showed both expression of ECD- and ICD-HER2, the p95HER2-overexpressing cells expressed ICD-HER2 specifically [3, 10]. Cell viability analysis revealed that the MDA-MB-231-HER2 cells were sensitive to trastuzumab, while MDA-MB-231-p95HER2 cells exhibited trastuzumab resistance (Fig. 2i, Additional file 3: Figure S7). Following exposure to NCT-547, the expression and phosphorylation of HER2 or p95HER2 were dramatically decreased in the HER2- and p95HER2-overexpressing cells, respectively (Fig. 2j). Ubiquitination appeared to be involved in the degradation of p95HER2, with immunocytochemical analysis revealing co-localization between p95HER2 and ubiquitin at the plasma membrane expressed as yellow signal (Fig. 2k). Forced expression of either HER2 or p95HER2 elevated STAT3 activity and increased its downstream signaling in TNBC cells (Additional file 3: Figure S8a). STAT3 plays major roles in migration and metastasis during breast cancer progression [6]. NCT-547 suppressed STAT3 activation, evidenced by marked downregulation of the STAT3 signaling-related factors, survivin and cyclin D1, as well as impairment of migratory capacity in both HER2- and p95HER2-overexpressing cells (Additional file 3: Figure S8b and S9). Furthermore, cell migration by trastuzumab-resistant JIMT-1 cells was also evidently reduced in the presence of NCT-547 (Additional file 3: Figure S10). These findings suggest that NCT-547 may have clinical applications in suppressing metastasis by trastuzumab-resistant HER2-positive breast cancers.

### NCT-547 inhibits tumor growth of trastuzumab-resistant JIMT-1 xenografts

**Fig. 3:**
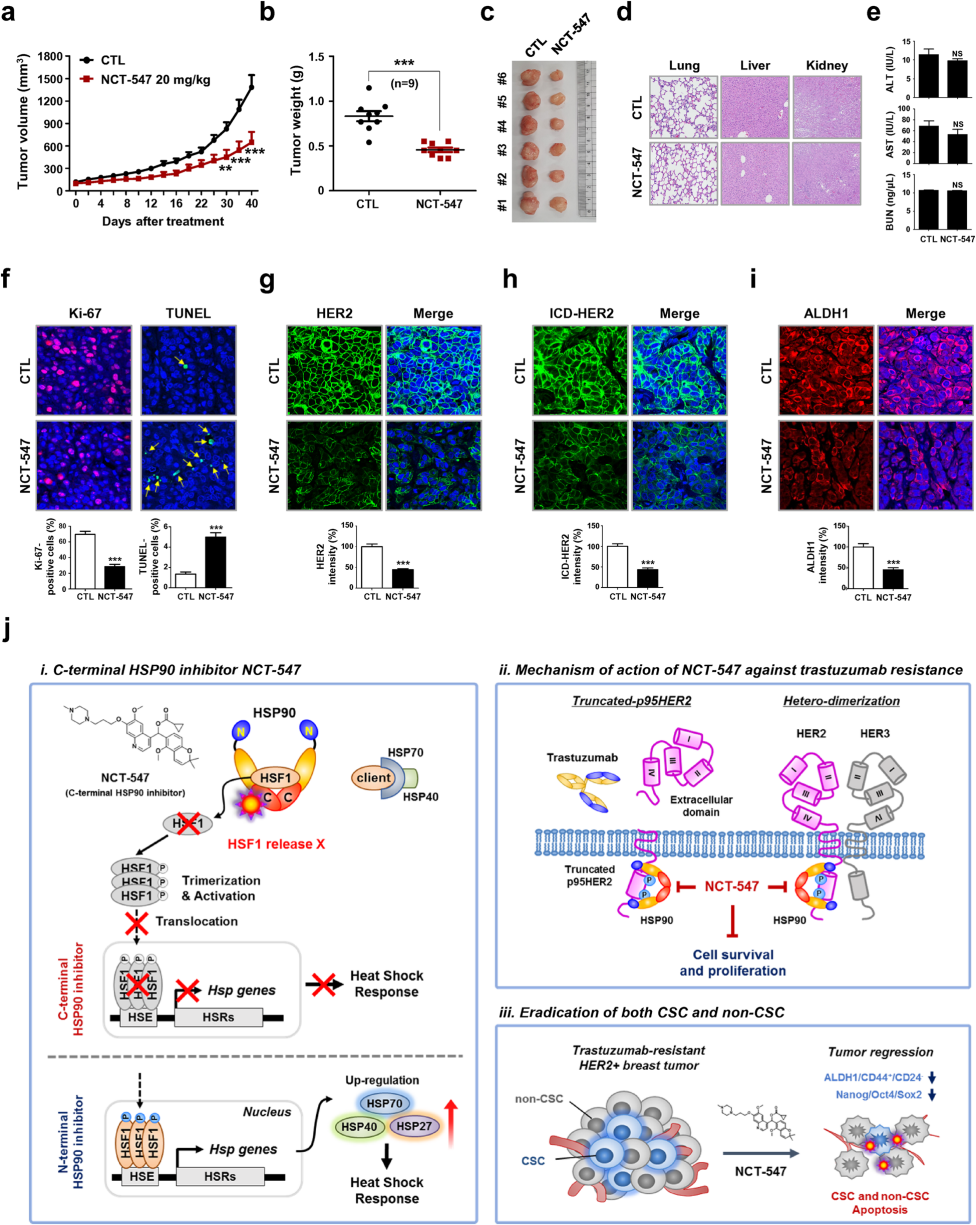
NCT-547 administration inhibits tumor growth in trastuzumab-resistant JIMT-1 xenografts in vivo. **a** JIMT-1 cells (3 × 10^6^) were injected into the animals and tumor volumes were measured with a caliper every other day for up to 40 days. Data were analyzed by two-way ANOVA followed by Bonferroni’s post hoc test (**p < 0.01). **b-c** Tumor weights and images from the control and NCT-547 treated groups (***p < 0.001). **d-e** Histological changes were examined in multiple organs (H&E staining: lung, liver and kidney), and hepatic and renal function were assessed via ALT/AST activity and BUN concentrations in the animal serum (NS; not significant). AST: aspartate aminotransferase; ALT: alanine aminotransferase; BUN: blood urea nitrogen. **f** Effect of NCT-547 on proliferating tumor cells was examined by Ki-67 staining. Tissue sections were stained for Ki-67 (red) and DAPI (blue), and Ki-67-positive cells were counted (***p < 0.001). Apoptosis induction following treatment with NCT-547 was determined by TUNEL assay (***p < 0.001). **g-h** NCT-547 treatment resulted in marked reductions of HER2 (**g**) and ICD-HER2 (**h**) in vivo, as determined by immunostaining of full-length HER2 and ICD-HER (***p < 0.001). **i** NCT-547-treated mice showed reduced expression of ALDH1 (***p < 0.001). All images were taken with a confocal microscope (original magnification: × 500). The fluorescence intensities were analyzed using a histogram tool in the Carl Zeiss software package. Data was analyzed by unpaired Student’s t-test. **j** Hypothetical models of i) Mode of action of NCT-547 is targeting of the C-terminal domain of HSP90 without induction of HSR. N-terminal HSP90 inhibitor activates HSF-1 to induce translocation into the nucleus and binding to HSEs, resulting in increased transcription of HSPs, such as HSP27, HSP40, and HSP70 which contribute to the attenuation of apoptosis. ii) NCT-547 degrades truncated p95HER2 and HER family members, and disturbs hetero-dimerization. iii) NCT-547 eradicates both proliferating tumor cells and cancer stem cells. [Heat shock response, HSR; Heat shock factor-1, HSF-1, Heat shock element, HSE]

To confirm the physiological relevance of our in vitro observations, we examined the impact of NCT-547 on tumor growth in trastuzumab-resistant xenografts. The growth of JIMT-1 tumors was significantly inhibited by treatment with NCT-547, and tumor burden in the NCT-547-treated group was less than the control counterparts (***p < 0.001, Fig. 3a-c). There were reduced numbers of Ki-67-positive cells and an increase in TUNEL-positive cells (***p < 0.001, Fig. 3f, Additional file 3: Figure S12). Inhibition of the tumor angiogenesis was evidenced by significant reductions in CD31-positive microvessels in both intratumoral and peritumoral areas (Additional file 3: Figure S13). Comparable with the in vitro findings, NCT-547 elicited a marked reduction in HER2 and ICD-HER2, with a decrease in ALDH1 expression also observed (***p < 0.001, Fig. 3g-i, Additional file 3: Figure S14 and S15).

There was no significant decline in body weight observed (Additional file 3: Figure S11). Moreover, our initial findings suggest that hepatic and renal health through the levels of AST, ALT, or BUN are relatively unaffected by NCT-547 with no histological findings in tissue sections (NS, Fig. 3d and e). Further investigation of the long-term safety profile for clinical application is planned.

## Conclusion

Our observations of the novel rationally-designed C-terminal HSP90 inhibitor NCT-547 suggests that it may have potential to address limitations in the treatment of trastuzumab-resistant HER2-positive breast cancer (Fig. 3j). Further profiling of this promising compound is warranted.

## Supplementary Information

**Additional file 1.** Materials and Methods.

**Additional file 2.** Experimental Procedure for the Synthesis of NCT-547.

**Additional file 3.** Supplementary Figures.

## Data Availability

All data generated or analyzed during this study are included either in this article or in the supplementary information files.

## Abbreviations

HER2: Human epidermal growth factor receptor 2
EGFR: Epidermal growth factor receptor
HSP: Heat shock protein
HSR: Heat shock response
HSF-1: Heat shock factor-1
HSEs: Heat shock elements
TNBC: Triple-negative breast cancer
PI: Propidium iodide
DMSO: Dimethyl sulfoxide
STR: Short tandem repeat
DEAB: Diethylamino-benzaldehyde
EC: Electrostatic complementarity
bFGF: Basic fibroblast growth factor
hEGF: Human epidermal growth factor
BSA: Bovine serum albumin
AST: Aspartate aminotransferase
ALT: Alanine aminotransferase
BUN: Blood urea nitrogen
SAR: Structure–activity relationship
CSCs: Cancer stem cells
BCSC: Breast cancer stem cell
CD31: Cluster of differentiation 31
MVD: Microvessel density
TUNEL: Terminal deoxynucleotidyl transferase dUTP nick end labeling
CD44: Cluster of differentiation 44
ALDH1: Aldehyde dehydrogenase 1
MFP: Mammary fat pad
PARP: Poly (ADP-ribose) polymerase
ICD: Intracellular domain
ECD: Extracellular domain
H&E: Hematoxylin and eosin
IP: Immunoprecipitation
IB: Immunoblot
IgG: Immunoglobulin G
Gelda: Geldanamycin
CTL: Control
