# Supplementary material for: A novel HSP90 inhibitor targeting the C-terminal domain attenuates trastuzumab resistance in HER2-positive breast cancer
Source: Mol Cancer. 2020 Nov 20;19:161. doi: 10.1186/s12943-020-01283-6 (PMC7678296; doi:10.1186/s12943-020-01283-6)
Supplement: Supplementary file 1 — Additional file 1. Materials and Methods. [file 12943_2020_1283_MOESM1_ESM.docx]

**Additional file 1. Materials and Methods**

***Reagents and antibodies***

A detailed description of the synthesis of NCT-547 is described in the supplementary information. Triton X-100, propidium iodide (PI), corn oil and dimethyl sulfoxide (DMSO) were obtained from Sigma-Aldrich (St. Louis, MO). Phosphatase inhibitor and protease inhibitor cocktail tablets were purchased from Roche Applied Sciences (Penzberg, Germany). The antibodies that were used include: STAT3, Ki-67, CD31, ALDH1A1, ICD-HER2 (CB11) (Abcam, MA); ICD-HER2 (4B5) (Ventana Medical Systems, AZ, USA); HER2, phospho-HER2 (Tyr1221/1222), HER3, phospho-HER3 (Tyr1289), EGFR, phospho-EGFR, AKT, phospho-STAT3 (Tyr705), vimentin, Nanog, Oct4, Sox2, PARP, cleaved-PARP, cleaved-caspase-3, cleaved-caspase-7, (Cell Signaling, CA); survivin, HSP70, HSP90, HSF-1, cyclin D1 (Santa Cruz Biotechnology, CA); GAPDH, β-actin (Sigma-Aldrich, MO). The secondary antibodies were horseradish peroxidase (HRP)-conjugated anti-rabbit and mouse IgG (Bio-Rad Laboratories, CA); and Alexa Fluor-488 and -594 goat anti-mouse IgG (Invitrogen, CA).

***Breast cancer cell culture***

The human breast cancer cell lines SKBR3, BT474, MDA-MB-453 (American Type Culture Collection), JIMT-1 (DSMZ GmbH, Germany), MDA-MB-231 (PerkinElmer, Inc. CT) and the normal human embryonic kidney cell line HEK293 (Korean Cell Line Bank) were cultured either in RPMI1640, MEM or DMEM (Gibco, MD) containing 10% fetal bovine serum (FBS) and streptomycin-penicillin (100 U/ml). The normal human mammary epithelial cell line MCF10A (ATCC) was cultured in Mammary Epithelial Cell Growth Basal Medium (MEBM), including hEGF, insulin, hydrocortisone and bovine pituitary extract (SingleQuotsTM Kit, Lonza, CA) containing streptomycin-penicillin (100 U/ml). Cells were incubated at 37°C in an atmosphere of 5% CO2. All cell lines were authenticated by short tandem repeat (STR) profiling by Macrogen Inc (Seoul, South Korea).

***Stabilized HER2 and p95HER2 overexpression in MDA-MB-231 cells***

HER2- and p95HER2-overexpressing MDA-MB-231 cells were generated using a lentiviral system according to the previously described method [[1](#_ENREF_1)]. Briefly, the HER2 or p95HER2 gene was amplified by PCR using specific primers [[1](#_ENREF_1)], and then inserted into a dual promoter lentivector (CD550A-1, System Biosciences, USA). Pseudoviral particles containing cDNA of full-length HER2 and p95HER2 were collected and concentrated via centrifugation before transfection into MDA-MB-231 target cells. After the infection, puromycin selection was performed and single colonies were isolated from a dish. The final concentration of puromycin was 3 μg/ml, and no mycoplasmas were detected in the resultant cell lines.

***Cell viability assay***

Cell viability assays were performed using a CelTiter 96* Aqueous One Solution Cell Proliferation Assay [MTs,3-(4,5-dyymethylthiazol-2-yl)-5-(3-carboxymethoxyphenyl)-2-(4-sulfophenyl)-2H-tetrazolium] (Promega, Madison, WI) according to the manufacturer’s instructions. The quantity of formazan product was measured via the absorbance at 490nm with a Spectramax Plus 384 microplate analyzer (Molecular Devices, CA)

***HSP90α (C-Terminal) inhibitor screening assay***

HSP90α (C-Terminal) Inhibitor Screening Assay Kit (#50317) was obtained from BPS Bioscience (CA, USA). All assays were carried out with an Optiplate-384 (PerkinElmer #6007290) and measured using an AlphaScreen® microplate reader. Diluted HSP90α (1.5 ng/µl) and PPID (10 ng/µl) with NCT-547, novobiocin, geldanamycin, and deguelin, were loaded in the Optiplate along with Detection alphaLISA® Acceptor Beads and Streptavidin-conjugated donor beads in accordance with the manufacturer’s guide. HSP90α:PPID binding activity with each HSP90 inhibitor was analyzed with a Varioskan LUX™ multimode microplate reader (Thermo Fisher Scientific, Rockford, IL)

***Flow cytometric cell death assay***

Cells were harvested and fixed with 95% ethanol containing 0.5% Tween-20 for 24 h before washing with PBS and staining with PI (50 µg/ml) and RNase (50 µg/ml) for 30 min. Stained cells were analyzed by flow cytometry using a Beckman Coulter FC 500 (Beckman Coulter, Brea, CA) and BD LSRFortessa™ X-20 Cell Analyzer (BD Biosciences, Piscataway, NJ).

**Aldefluor-positivity assay and CD24/44 staining**

An Aldefluor assay kit (Stemcell Technology, Vancouver, BC) was used to evaluate ALDH1 activity according to the manufacturer’s protocol. Cells were incubated at 37°C for 45 min in Aldefluor assay buffer containing the ALDH protein substrate BODIPY-aminoactaldehyde BAAA, 1 µM / 0.5 × 10^6^ cells. 50mM of diethylamino-benzaldehyde (DEAB) as a specific inhibitor of ALDH1 was used to define the Aldefluor-positive population. For CD24/44 staining, cells were stained with FITC- and PE-conjugated anti-mouse IgG, and either FITC-conjugated anti-CD24 or PE-conjugated anti-CD44 (BD Biosciences). Aldefluor- and CD24/44-stained cells were analyzed with a Beckman Coulter FC 500 flow cytometer system (Beckman Coulter) and BD LSRFortessa™ X-20 Cell Analyzer (BD Biosciences).

***Immunoblot analysis***

Cells were solubilized in lysis buffer [30 mM NaCl, 0.5% Triton X-100, 50 mM Tris-HCl (pH 7.4)] containing phosphatase and protease inhibitor cocktail tablets. Supernatant was collected after centrifugation (14,000 g, 4°C, 20 min) and protein concentrations were measured with a Bradford protein assay kit (Bio-Rad Laboratories). Equal quantities of protein (30 μg) were subjected to SDS-PAGE and electrotransferred onto a nitrocellulose membrane (GE Healthcare Life Sciences, Buckinghamshire, UK). The membranes were incubated overnight at 4°C with primary antibodies diluted in 5% BSA [HER2 (1:2000), p-HER2 (1:2000), HER3 (1:2000), p-HER3 (1:2000), EGFR (1:3000), p-EGFR (1:2000), HSP90 (1:2000), HSP70 (1:2000), Akt (1:2000), Survivin (1:1000), Pro-PARP (1:2000), cleaved-PARP (1:1000), cleaved-caspase-7 (1:1000), cleaved-caspase-3 (1:1000), Nanog (1:1000), Oct4 (1:1000), Sox2 (1:1000), Actin (1:30,000), and GAPDH (1:3000)], followed by incubation with HRP-conjugated anti-rabbit and mouse IgGs (1:3000-1:10000). Signal intensity was detected using an Enhanced Chemiluminescence Kit (Thermo Scientific Inc., Rockford, IL) and x-ray film (Agfa Healthcare, Mortsel, Belgium) and quantitated using AlphaEaseFC software (Alpha Innotech, San Leandro, CA).

***Immunoprecipitation***

Cells were lysed in Pierce immunoprecipitation cell solubilizing buffer (Thermo Fisher Scientific) containing a cocktail of phosphatase and protease inhibitors. Supernatant was collected after centrifugation of 15,000 rpm, 4°C, 20 min. Equal quantities were incubated with 10µg of rabbit polyclonal HER2 antibody conjugated to Dynabeads (Invitrogen) at 4°C overnight. The beads were washed with washing buffer, boiled with 2x loading sample buffer for 5 min and loaded for SDS-PAGE. Immunoblotting for EGFR, HER2, HER3, and HSP90 was then conducted.

***Immunocytochemistry***

The procedures were performed as previously described [[2](#_ENREF_2)]. The cells with primary antibodies in antibody-diluent (Dako, Glostrup, Denmark) were incubated overnight at 4°C. For secondary antibody reactions, Alexa Fluor®-594 or -488 conjugated secondary antibodies (Invitrogen, Carlsbad, CA) were used for staining before counterstaining with ProLong Gold Antifade Reagent with DAPI (Life Technologies, Carlsbad, CA). Images were acquired using a Carl Zeiss confocal microscope (Weimar, Germany), and the intensity of the images was analyzed using the intensity profile tool.

***Molecular Modeling and Docking Analysis***

A docking simulation was performed using the Tripos Sybyl-X 2.1 [[3](#_ENREF_3)] in the Windows 7 operating system. Molecular structures of ligands (R- and S-enantiomer of NCT-547) were prepared using the sketch module in Sybyl. After each sketch, charge and energy minimization were assigned to all atoms of the ligands using the molecule minimize module. Tripos force field and Gasteiger-Hückel charge, a conjugate-gradient method with a convergence criterion of 0.001 kcal mol-1.Å-1, and max iteration to 10000 for energy minimization were used. For the receptor, a previously reported homology model of open conformation of hHSP90 homodimer-ATP complex [[4](#_ENREF_4), [5](#_ENREF_5)] was used. Protein structures were refined using the structure preparation tool module, and Kollman All charge was assigned. The protomol, a computational description of the binding site at which putative ligands are aligned, was defined as 20 amino acids adjacent to ATP in the C-terminal region with a threshold parameter of 0.50 and a bloat parameter of 5 Å. Docking was conducted with the default settings of Surflex-Dock GeomX, generating 50 maximum poses per ligand and with CScore (consensus score) calculations [[6](#_ENREF_6)]. The binding conformation of NCT-547 was selected by considering the Surflex-Dock score and CScore, and visual inspection. The S-form of NCT-547 formed favorable interactions with the receptor, and its complex was selected as the final docking model.

Visualization and rendering of docking models were performed on a Maestro graphic user interface in Schrödinger 2020-1 [[7](#_ENREF_7)] and Benchware 3D explorer program [[8](#_ENREF_8)]. To calculate the electrostatic complementarity (EC) of ligand-protein complexes, the coordinates of ATP- and NCT-547-bound hHSP90 were exported as a mol2 file to the Flare program [[9](#_ENREF_9), [10](#_ENREF_10)]. EC surface and scores between ligands and the receptor were generated using default settings.

***Animals, xenograft experiments***

All animal procedures were carried out in accordance with animal care guidelines approved by Korea University. 5-week-old female BALB/c nude mice were purchased from Shizuoka Laboratory Animal center (Shizuoka, Japan) and housed in a specific pathogen-free environment for one week before the study. JIMT-1 cells (3 × 10^6^) were injected into the mammary fat pads of 6-week-old BALB/c nude female mice (n = 9/each group). After 1 week, solvent control (1:9 DMSO:corn oil mixture) and NCT-547 (20 mg/kg) were administered intraperitoneally every other day for up to 40 days. Tumor volumes and body weight were calculated using the formula V = (Length × Width2)/2.

***Immunohistochemistry and in-situ localization of apoptosis (TUNEL)***

At sacrifice, the tumors were removed, fixed in 4% paraformaldehyde, and embedded in paraffin. Tissue sections of 4-µm thickness were mounted on glass slides and then deparaffinized with xylene and dehydrated through a graded alcohol series to water. For antigen retrieval, sections were boiled in citric acid buffer (pH 6.0). Tissue sections were incubated with primary antibodies (Ki-67; 1:100, HER2; 1:100, and 4B5 ICD-HER2; 1:100) at 4°C overnight. For secondary antibody reactions, Alexa Fluor®-594 or -488 conjugated secondary antibodies (Invitrogen, Carlsbad, CA) were applied to tissue sections for staining, incubated at RT for 2 h, and mounted with ProLong Gold Antifade Reagent with DAPI (Life Technologies, Carlsbad, CA). TUNEL assays were performed using a TUNEL kit (Roche Applied Sciences, Penzberg, Germany) according to the manufacturer’s protocol.

***In vitro and in vivo mammosphere formation assays***

For the in vitro mammosphere-forming assay, cells were plated in ultralow attachment dishes (Corning, NY) and HuMEC basal serum-free medium (Gibco), supplemented with B27 (1:50, Invitrogen, Carlsbad, CA), 20 ng/ml basic fibroblast growth factor (bFGF, sigma-Aldrich), 20 ng/ml human epidermal growth factor (hEGF, sigma-Aldrich), 5 µg/ml heparin, 1% antibiotic-antimycotic, and 15 µg/ml, gentamycin at 37°C in an atmosphere of 5% CO2. For the in vivo assay, xenografted tumors were prepared as per the animal model described in Animals, xenograft experiments. The tumors were harvested when volumes reached 200-250 mm3 and were dissociated with type III collagenase (2 mg/ml) until the tissues were digested into single cells. The resultant single cells were filtered through a 40 µm cell strainer, centrifuged at 200 g for 5 min and washed with medium containing 0.2% bovine serum albumin (BSA). The cells were seeded in ultralow attachment dishes and cultured under the same conditions as for the in vitro protocol. The numbers and volumes of mammospheres were measured under an Olympus CKX53 inverted microscope.

***Wound healing assay***

For kinetic migration analysis, JIMT-1, MDA-MB231-HER2 and MDA-MB-231-p95HER2 cells were seeded to ~80% confluency in 96-well plates, respectively (Essen ImageLock, Essen Biosciences, Ann Arbor, MI, USA). Wound areas were made with a 96-pin Wound Maker device and washed with PBS to prevent reattachment of dislodged cells. Cells were treated with NCT-547 immediately after wound scratching, and images of the scratched fields were automatically acquired and registered every hour up to 72 h with an IncuCyte™ ZOOM® Kinetic Imaging System. The relative wound density was analyzed using the IncuCyte™ Scratch Wound Cell Migration Software Module.

***Serum biochemistry profiles for biomarkers of liver and renal injury***

At sacrifice, blood samples of each animal were collected and serum samples were acquired by centrifuging at 3000 rpm for 20 min. Serum enzyme activities of aspartate aminotransferase (AST) and alanine aminotransferase (ALT), and blood urea nitrogen (BUN) levels were determined with an AST, ALT and BUN assay kit following the manufacturer’s protocol (Sigma-Aldrich, Louis, MO).

***Statistical analysis***

Statistical analysis was performed using GraphPad Prism 5.0 statistical software (San Diego, CA). The results are expressed as mean values ± SEM of at least three independent experiments. Data were analyzed by Student’s t-test and one- or two-way ANOVA, as appropriate. Two-way ANOVA was used to evaluate the effects and interactions of two variables, and multiple comparisons were made using Bonferroni’s post hoc test. p values of <0.05 were considered statistically significant.

**References**

1. Pedersen K, Angelini PD, Laos S, Bach-Faig A, Cunningham MP, Ferrer-Ramon C, Luque-Garcia A, Garcia-Castillo J, Parra-Palau JL, Scaltriti M, et al: **A naturally occurring HER2 carboxy-terminal fragment promotes mammary tumor growth and metastasis.** *Mol Cell Biol* 2009, **29:**3319-3331.

2. An H, Kim JY, Oh E, Lee N, Cho Y, Seo JH: **Salinomycin Promotes Anoikis and Decreases the CD44+/CD24- Stem-Like Population via Inhibition of STAT3 Activation in MDA-MB-231 Cells.** *PLoS One* 2015, **10:**e0141919.

3. **Certara, L. P. SX. 2.1.1 ed. SYBYL molecular modeling software, St. Louis, MO, 2013.**

4. Lee SC, Min HY, Choi H, Kim HS, Kim KC, Park SJ, Seong MA, Seo JH, Park HJ, Suh YG, et al: **Synthesis and Evaluation of a Novel Deguelin Derivative, L80, which Disrupts ATP Binding to the C-terminal Domain of Heat Shock Protein 90.** *Mol Pharmacol* 2015, **88:**245-255.

5. Sgobba M, Forestiero R, Degliesposti G, Rastelli G: **Exploring the binding site of C-terminal hsp90 inhibitors.** *J Chem Inf Model* 2010, **50:**1522-1528.

6. Clark RD, Strizhev A, Leonard JM, Blake JF, Matthew JB: **Consensus scoring for ligand/protein interactions.** *J Mol Graph Model* 2002, **20:**281-295.

7. **Schrödinger Release 2020-4: Schrödinger, LLC, New York, NY, 2019.**

8. **Certara, L. P., Benchware 3D explorer 2.8, St. Louis, MO, 2014.**

9. **Flare, version 3.0, Cresset®, Litlington, Cambridgeshire, UK;** [**http://www.cresset-group.com/flare/**](http://www.cresset-group.com/flare/)**.**

10. Bauer MR, Mackey MD: **Electrostatic Complementarity as a Fast and Effective Tool to Optimize Binding and Selectivity of Protein-Ligand Complexes.** *J Med Chem* 2019, **62:**3036-3050.
