# Supplementary material for: A novel HSP90 inhibitor targeting the C-terminal domain attenuates trastuzumab resistance in HER2-positive breast cancer
Source: Mol Cancer. 2020 Nov 20;19:161. doi: 10.1186/s12943-020-01283-6 (PMC7678296; doi:10.1186/s12943-020-01283-6)
Supplement: Supplementary file 2 — Additional file 2. Experimental Procedure for the Synthesis of NCT-547. [file 12943_2020_1283_MOESM2_ESM.docx]

**Additional file 2. Experimental Procedure for the Synthesis of NCT-547**

**General.**

All chemical reagents were commercially available. Melting points were determined on a melting point Buchi B-540 apparatus and are uncorrected. Silica gel column chromatography was performed on silica gel 60, 230–400 mesh, Merck. The preparative layer chromatography (PLC) plates used PLC silica gel 60 F254, 1 mm, Merck. 1H NMR spectra were recorded on a JEOL JNM-LA 300 at 300 MHz and Bruker Analytik, DE/AVANCE Digital 400 at 400 MHz. Chemical shifts are reported in ppm units with Me4Si as a reference standard. Mass spectra were recorded on a VG Trio-2 GC−MS instrument and a 6460 Triple Quad LC−MS instrument.

**5-Hydroxy-2,2-dimethyl-2H-chromene-6-carbaldehyde (2).**

A solution of commercially available 2,4-dihydroxybenzaldehyde (**1**) (27.36 g, 198.1 mmol) in pyridine (50 mL) was treated with 3-methyl-2-butenal (22.8 mL, 237.7 mmol). After being stirred at 140 °C overnight, the reaction mixture was diluted with EtOAc, washed by 1 N HCl, dried over MgSO_4_, and concentrated in vacuo. The residue was purified by flash column chromatography on silica gel to afford **2** (12.94 g) as a yellow solid in 32% yield: ^1^H-NMR (300 MHz, CDCl_3_) δ 11.62 (s, 1H), 9.63 (s, 1H), 7.26 (d, *J* = 8.43 Hz, 1H), 6.66 (d, *J* = 10.05 Hz, 1H), 6.40 (dd, *J* = 8.61 and 0.72 Hz, 1H), 5.58 (d, 10.08 Hz, 1H), 1.49 (s, 6H).

**5-Methoxy-2,2-dimethyl-2H-chromene-6-carbaldehyde (3).**

A solution of **2** (12.94 g, 63.4 mmol) in DMF (30 mL) was treated with K_2_CO_3_ (26.25 g, 190.2 mmol) followed by iodomethane (4.34 mL, 69.74 mmol). After being stirred at 70 °C for 3 h, the reaction mixture was cooled to room temperature. The mixture was extracted with EtOAc several times and the combined organic layers was washed with brine, dried over MgSO_4_, and concentrated in vacuo. The residue was purified by flash column chromatography on silica gel with EtOAc/hexanes (1:4) as eluant to give **3** (10.09 g) as a yellow oil in 73% yield: ^1^H-NMR (300 MHz, CDCl_3_) δ 10.15 (s, 1H), 7.63 (d, *J* = 8.58 Hz, 1H), 6.62 (d, *J* = 8.61 Hz, 1H), 6.57 (d, *J* = 10.08 Hz, 1H), 5.67 (d, *J* = 10.08 Hz, 1H), 3.88 (s, 3H), 1.44 (s, 6H).

**1-(3-Methoxy-4-(methoxymethoxy)phenyl)ethan-1-one (5).**

A solution of commercially available acetovanillone (**4**) (5 g, 29.6 mmol) in DMF (20 mL) was treated slowly with NaH (1.3 g, 32.6 mmol) at 0 °C, stirred for 30 min and then added dropwise with the chloromethyl methyl ether (2.7 mL, 35.5 mmol). After being stirred at 0 °C for 3 h, the reaction mixture was diluted with water and extracted with EtOAc several times. The combined organic layers was washed with brine, dried over MgSO4 and concentrated in vacuo to give **5** (4.86 g) in 77% yield. The residue was used for the next step without further purification: ^1^H-NMR (400 MHz, DMSO-d_6_) δ 7.58 (dd, *J* = 8.40 and 2.00 Hz, 1H), 7.47 (d, *J* = 2.00 Hz, 1H), 7.15 (d, *J* = 8.40 Hz, 1H), 5.26 (s, 2H), 3.83 (s, 3H), 3.39 (s, 3H), 2.53 (s, 3H).

**1-(5-Methoxy-4-(methoxymethoxy)-2-nitrophenyl)ethan-1-one (6).**

A cooled solution of **5** (4.86 g, 23.1 mmol) in acetonitrile (20 mL) at -10 °C was treated with NH_4_NO_3_ (4.62 g, 57.7 mmol) followed by trifluoroacetic anhydride (8.08 mL, 57.8 mmol) and stirred until the material was consumed. The mixture was warmed up to 0 °C and quenched by saturated NaHCO_3_ solution. The resulting mixture was extracted with EtOAc and the organic layer was washed with brine, dried over MgSO_4_ and concentrated in vacuo. The residue was purified by flash column chromatography on silica gel to give **6** (5.8 g) in 98% yield as a yellow solid: ^1^H-NMR (400 MHz, CDCl_3_) δ 7.90 (s, 1H), 6.76 (s, 1H), 5.29 (s, 2H), 3.96 (s, 3H), 3.50 (s, 3H), 2.47 (s, 3H).

**1-(2-Amino-5-methoxy-4-(methoxymethoxy)phenyl)ethan-1-one (7).**

A solution of KOH (6.37 g, 113.5 mmol) in H_2_O (30 mL) was treated dropwise with formic acid (8.6 mL, 227 mmol) and stirred at room temperature for 1 h. The mixture was treated with **6** (5.8 g, 22.7 mmol) in MeOH (20 mL) followed by Pd/C (500 mg) and the stirred at ambient temperature overnight. The reaction mixture was filtered through celite and washed with MeOH. The resulting solution was evaporated in vacuo, suspended in water and neutralized by 1 N KOH solution. The residue was extracted by EtOAc and the organic layer was dried over MgSO_4_ and concentrated in vacuo. The residue was purified with flash column chromatography on silica gel with EtOAc/hexanes (1:3) as eluant to give **7** (3.81 g) in 74% yield as brown solid: ^1^H-NMR (400 MHz, CDCl_3_) δ 7.11 (s, 1H), 6.42 (s, 1H), 6.13 (br, 2H), 5.23 (s, 2H), 3.81 (s. 3H), 3.48 (s, 3H), 2.50 (s, 3H).

**6-Methoxy-7-(methoxymethoxy)quinolin-4-ol (8).**

A solution of **7** (3.81 g, 16.9 mmol) in ethyl formate (20 mL) was treated with sodium ethoxide (21% w/w in EtOH) (50 mL, 132.8 mmol) and was refluxed overnight. The reaction mixture was evaporated in vacuo, diluted with water, neutralized with 1 N HCl and extracted with EtOAc several times. The organic layers was washed with brine dried over MgSO_4_ and concentrated in vacuo. The residue was purified by flash column chromatography on silica gel with MeOH/EtOAc (1:4) as eluant to give **8** (2.21 g) in 56% yield as slight yellow solid: ^1^H-NMR (400 MHz, CD_3_OD) δ 7.87 (d, *J* = 6.80 Hz, 1H), 7.65 (s, 1H), 7.27 (s, 1H), 6.29 (d, *J* = 7.20 Hz, 1H), 5.34 (s, 2H), 3.95 (s, 3H), 3.51 (s, 3H).

**4-Bromo-6-methoxy-7-(methoxymethoxy)quinolone (9).**

A cooled solution of **8** (2.21 g, 9.39 mmol) in DMF (20 mL), at 0 °C was treated dropwise with PBr_3_ (1.07 mL, 11.3 mmol) and stirred at room temperature for 3 h. The mixture was cooled down to 0 °C, quenched with K_2_CO_3_, diluted with water and extracted with EtOAc. The organic layer was dried over MgSO_4_ and concentrated in vacuo. The residue was purified with flash chromatography on silica gel with EtOAc/hexanes (1:3) as eluant to give **9** (2.62 g) in 93% yield as a slight yellow solid: ^1^H-NMR (400 MHz, CD_3_OD) δ 8.42 (d, *J* = 4.40 Hz, 1H), 7.71 (d, *J* = 5.20 Hz, 1H), 7.62 (s, 1H), 7.47 (s, 1H), 5.40 (s, 2H), 4.03 (s, 3H), 3.52 (s, 3H).

**4-Bromo-6-methoxyquinolin-7-ol (10).**

A solution of **9** (2.62 g, 8.79 mmol) in MeOH (20 mL) was treated with 4 N HCl (10.98 mL) and stirred at room temperature overnight. The mixture was evaporated in vacuo, dissolved with water, neutralized with 1 N K_2_CO_3_ and filtered to afford **10** (2.1 g) in 94% yield as a yellow solid: ^1^H-NMR (300 MHz, CD_3_OD) δ 8.30 (d, *J* = 4.95 Hz, 1H), 7.52 (d, *J* = 4.95 Hz, 1H), 7.37 (s, 1H), 7.28 (s, 1H), 4.03 (s, 3H).

**4-Bromo-7-(3-bromopropoxy)-6-methoxyquinoline (11).**

A solution of **10** (1.1 g, 4.33 mmol) and 18-crown-6 (1.14 g, 4.33 mmol) in DMF (20 mL) was treated with K_2_CO_3_ (2.39 g, 17.32 mmol) and stirred for 1 h at room temperature. Then the mixture was treated with 1,3-dibromopropane (1.32 mL, 13 mmol) and stirred at room temperature overnight. The reaction mixture was diluted with water and extracted with EtOAc several times. The combined organic layers was washed with brine, dried over MgSO_4_ and concentrated in vacuo. The residue was purified with flash chromatography on silica gel with EtOAc/hexanes (1:1) as eluant to give **11** (1.38 g) in 85% yield as a white solid: ^1^H-NMR (400 MHz, CDCl_3_) δ 8.45 (d, *J* = 4.80 Hz, 1Hz), 7.53 (d, *J* = 4.80 Hz, 1H), 7.42 (s, 1H), 7.36 (s, 1H), 4.31 (t, *J* = 6.00 Hz, 2H), 4.02 (s, 3H), 3.64 (t, *J* = 6.40 Hz, 2H), 2.45 (quint, *J* = 6.00 Hz, 2H).

**4-Bromo-6-methoxy-7-(3-(4-methylpiperazin-1-yl)propoxy)quinolone (12)**

A solution of **11** (1.38 g, 3.68 mmol) in DMF (20 mL) was treated with 1-methylpiperazine (0.82 mL, 7.36 mmol) followed by triethylamine (1.03 mL, 7.36 mmol). After being stirred at room temperature overnight, the reaction mixture was diluted with water and extracted with EtOAc several times. The combined organic layers was washed with brine, dried over MgSO_4_ and concentrated in vacuo. The residue was purified with flash chromatography on silica gel with MeOH/CH_2_Cl_2_ (1:4) as eluant to give **12** (1.07 g) in 74% yield as a slight yellow solid: ^1^H-NMR (400 MHz, CDCl_3_) δ 8.44 (d, *J* = 4.80 Hz, 1H), 7.52 (d, *J* = 5.20 Hz, 1H), 7.39 (s, 1H), 7.35 (s, 1H), 4.23 (t, *J* = 6.80 Hz, 2H), 4.02 (s, 3H), 2.56 (t, *J* = 6.80 Hz, 2H), 2.89-2.32 (br, 8H), 2.28 (s, 3H), 2.10 (quint, *J* = 6.80 Hz, 2H).

**(5-Methoxy-2,2-dimethyl-2H-chromen-6-yl)(6-methoxy-7-(3-(4-methylpiperazin-1-yl)propoxy)quinolin-4-yl)methanol (13).**

A cooled solution of **12** (300 mg, 0.81 mmol) in anhydrous THF (5 mL) at -78 °C was treated dropwise with n-BuLi (2.5 M in n-hexane, 0.39 mL, 0.972 mmol) followed by a slow solution of **3** (212 mg, 0.972 mmol) in anhydrous THF (1 mL). After being stirred for 3 h at -78 °C, the resulting solution was warmed to room temperature, quenched with saturated NH_4_Cl solution and extracted with EtOAc. The organic layer was washed with brine, dried over MgSO_4_, and concentrated in vacuo. The residue was purified by PLC with MeOH/CH_2_Cl_2_ (1:4) as the elutant to afford **13** (230 mg) in 53% yield as a slight yellow solid: ^1^H-NMR (400 MHz, CDCl_3_) δ 8.73 (d, *J* = 4.80 Hz, 1H), 7.65 (d, *J* = 4.80 Hz, 1H), 7.36 (s, 1H), 7.13 (s, 1H), 6.80 (d, *J* = 8.40 Hz, 1H), 6.62 (s, 1H), 6.57 (d, *J* = 9.60 Hz, 1H), 6.44 (d, *J* = 8.80 Hz, 1H), 5.65 (d, *J* = 10.00 Hz, 1H), 4.20-4.14 (m, 2H), 3.88 (s, 3H), 3.80 (s, 3H), 2.80-2.30 (m, 8H), 2.52 (t, *J* = 7.20 Hz, 2H), 2.27 (s, 3H), 2.05 (quint, *J* = 6.80 Hz, 2H), 1.38 (s, 6H).

**(5-Methoxy-2,2-dimethyl-2H-chromen-6-yl)(6-methoxy-7-(3-(4-methylpiperazin-1-yl)propoxy)quinolin-4-yl)methyl cyclopropanecarboxylate (NCT-547).**

A cooled solution of **13** (230 mg, 0.43 mmol) in CH_2_Cl_2_ (5 mL) at 0 °C were treated with pyridine (0.42 mL, 0.516 mmol) and cyclopropanecarbonyl chloride (0.47 mL, 0.516 mmol) and stirred overnight at room temperature. The reaction mixture was quenched with saturated NaHCO_3_ solution and extracted with CH_2_Cl_2_ several times. The organic layers were washed with water, dried over MgSO_4_, and concentrated in vacuo. The residue was purified by PLC with MeOH/CH_2_Cl_2_ (1:4) as the elutant to afford **NCT-547** (238 mg) in 92% yield as a white solid: mp: 43-45 ^o^C. ^1^H-NMR (400 MHz, CDCl_3_) δ 8.68 (d, *J* = 4.80 Hz, 1H), 7.74 (s, 1H), 7.39 (d, *J* = 5.20 Hz, 1H), 7.36 (s, 1H), 7.30 (s, 1H), 6.95 (d, *J* = 8.80 Hz, 1H), 6.53 (d, *J* = 10.00 Hz, 1H), 6.48 (d, *J* = 8.80 Hz, 1H), 5.63 (d, *J* = 10.00 Hz, 1H), 4.22-4.13 (m, 2H), 3.87 (s, 3H), 3.83 (s, 3H), 2.89-2.44 (m, 8H), 2.58 (t, *J* = 6.80 Hz, 2H), 2.39 (s, 3H), 2.06 (quint, *J* = 6.80 Hz, 2H), 1.80-1.74 (m, 1H), 1.39 (s, 6H), 1.08-1.00 (m, 2H), 0.96-0.88 (m, 2H). HRMS (FAB) calc. for C_35_H_43_N_3_O_6_ [M + H]^+^ 602.3230, found 602.3236.
