# Supplementary material for: A novel HSP90 inhibitor targeting the C-terminal domain attenuates trastuzumab resistance in HER2-positive breast cancer
Source: Mol Cancer. 2020 Nov 20;19:161. doi: 10.1186/s12943-020-01283-6 (PMC7678296; doi:10.1186/s12943-020-01283-6)
Supplement: Supplementary file 3 — Additional file 3. Supplementary Figures. [file 12943_2020_1283_MOESM3_ESM.docx]

**Additional file 3. Supplementary Figures**

***Supplementary Figure S1***

**Supplementary Figure S1.** Synthetic scheme of NCT-547

*Reagents and conditions*: a) 3-methyl-2-butenal, pyridine, 140 ^o^C, overnight; b) MeI, K_2_CO_3_, DMF, 70 ^o^C, 2 h; c) chloromethyl methyl ether, NaH, DMF, 0 ^o^C-r.t., 3 h; d) NH_4_NO_3_, (CF_3_CO)_2_O, MeCN, -10 ^o^C,1 h; e) KOH, HCOOH, Pd/C, MeOH, H_2_O, r.t, overnight; (f) NaOEt, ethyl formate, reflux, overnight; g) PBr_3_, DMF, 0 ^o^C-r.t., 3 h; h) HCl, MeOH, r.t., overnight; i) 1,3-dibromopropane, K_2_CO_3_, r.t., overnight; j) 1-methylpiperazine, TEA, DMF, r.t., overnight; k) n-BuLi, THF, -78 ^o^C before adding **3** in THF, 3 h; l) cyclopropanecarbonyl chloride, pyridine, CH_2_Cl_2_, 0 ^o^C-.r.t., overnight

***Supplementary Figure S2***


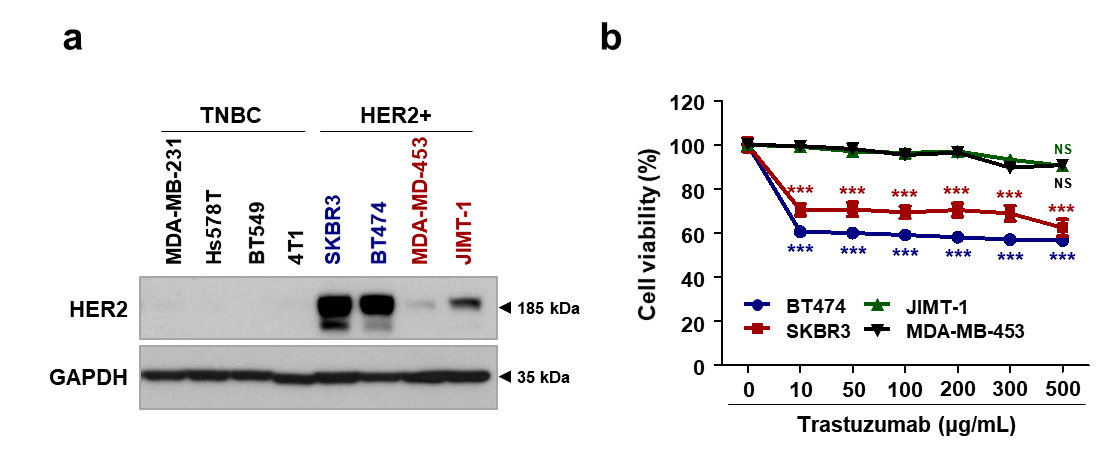


**Supplementary Figure S2**. HER2 expression and trastuzumab response in HER2-positive breast cancer cell lines

**a** Comparison of immunoblot analysis for basal levels of HER2 between trastuzumab-sensitive [SKBR3 and BT474] and -resistant [JIMT-1 and MDA-MB-453] HER2-positive breast cancer cells. TNBC [MDA-MB-231, Hs578T, BT549 and 4T1] cells exhibited no expression of HER2. GAPDH was used as a loading control. **b** Effect of trastuzumab (0-500 μg/ml, 72 h) on viability of HER2-positive breast cancer cells as determined by MTS assay (***p<0.001, NS). Trastuzumab-sensitive BT474 and SKBR3 cells exhibited greater sensitivity to trastuzumab, whereas JIMT-1 and MDA-MB-453 cells showed no significant response to trastuzumab. At least three independent experiments were performed and the data are shown as mean values ± SEM. Data were analyzed by One-way ANOVA followed by Bonferroni’s post hoc test. [Triple-negative breast cancer, TNBC]

***Supplementary Figure S3***

**
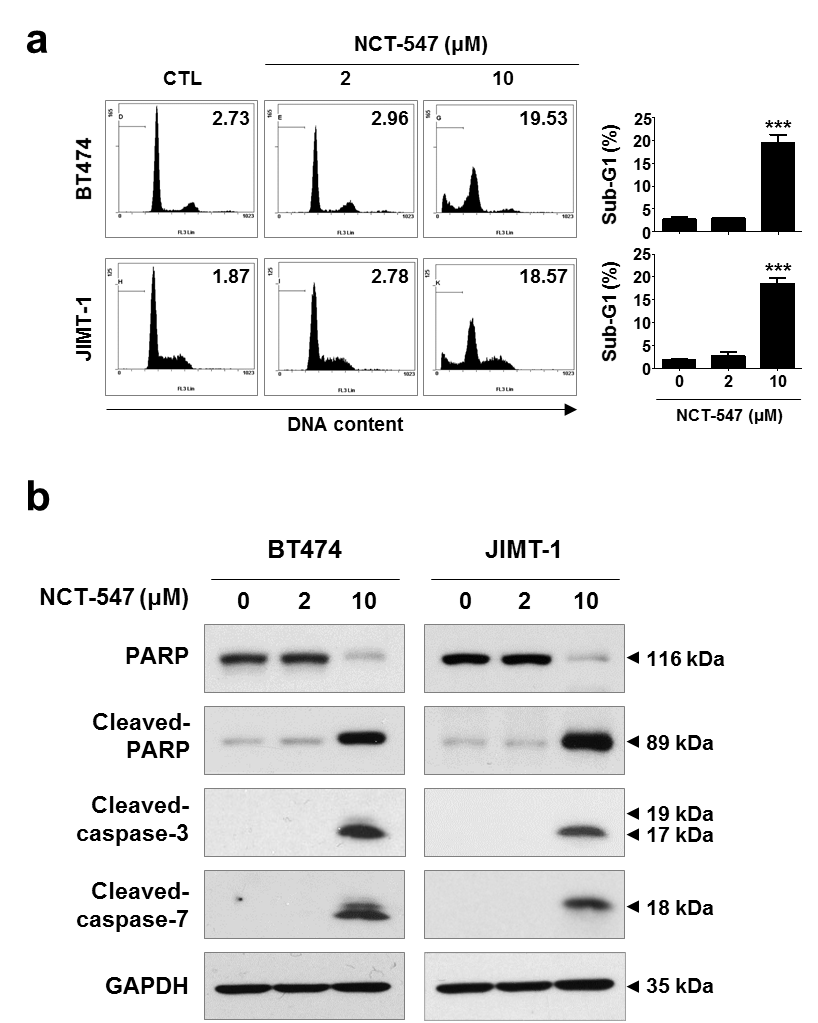
**

**Supplementary Figure S3.** NCT-547-induced apoptosis is mediated by caspase-3/-7 activation.

**a** Sub-G1 fractions of BT474 and JIMT-1 cells were estimated with flow cytometry (area under the grey horizontal bar) after treatment with NCT-547 (0-10 μM) for 72 h. The results are shown as mean ± SEM of at least three independent experiments (***p<0.001). **b** Expression of apoptotic factors PARP, cleaved-PARP, cleaved-caspase-3 and cleaved-caspase-7 in BT474 and JIMT-1 cells as determined by immunoblotting. GAPDH was used as a loading control.

***Supplementary Figure S4***


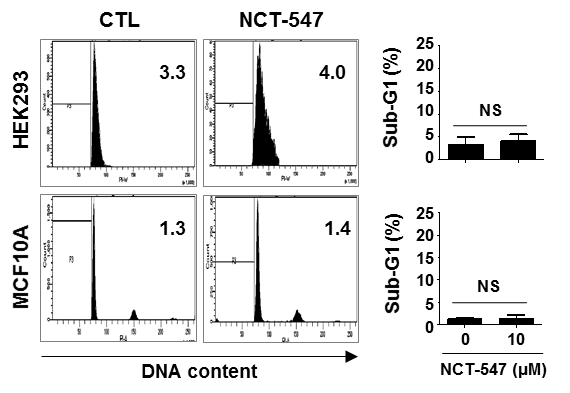


**Supplementary Figure S4.** NCT-547 does not affect apoptosis in non-malignant cells.

Normal human embryonic kidney HEK293 and normal human mammary epithelial MCF10A cells were treated with NCT-547 (10 µM) for 72 h. Apoptosis was analyzed by flow cytometry and the percentage of the sub-G1 population was quantified (NS; not significant). The results are shown as mean ± SEM of at least three independent experiments.

***Supplementary Figure S5***

**
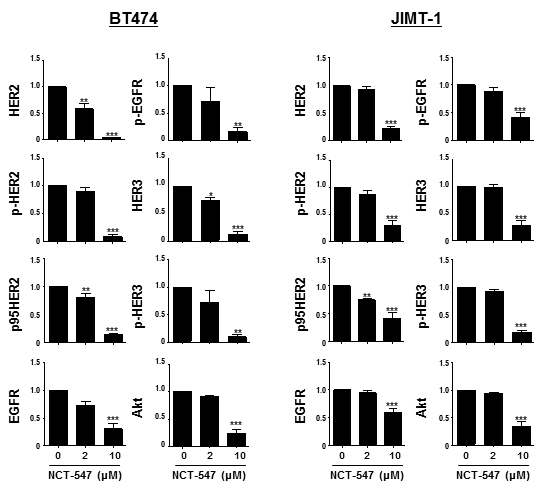
**

**Supplementary Figure S5.** NCT-547 downregulates the expression of HSP90 client proteins.

Quantitation of signal intensity of full-length p185HER2, aminoterminal-truncated p95HER2, phospho-HER2 (Tyr1221/1222), HER3, phospho-HER3 (Tyr1289), EGFR, phospho-EGFR (Tyr1068), and Akt relative to GAPDH expression, based on at least three independent experiments (*p<0.05).

***Supplementary Figure S6***

**
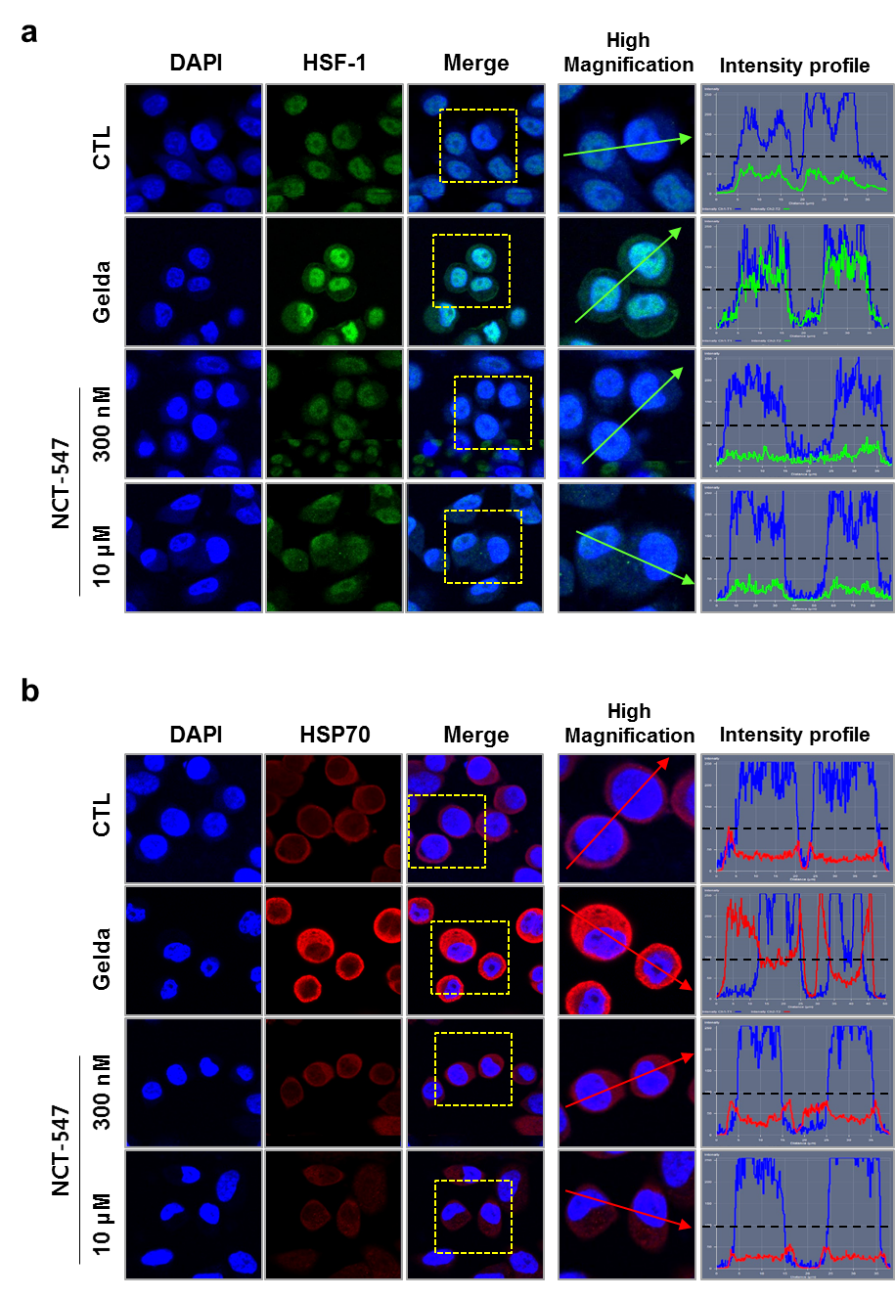
**

**Supplementary Figure S6.** NCT-547 does not induce the heat shock response.

**a-b** SKBR3 cells immunostained for HSF-1 (green, **a**) and HSP70 (red, **b**) with DAPI (blue) after exposure to NCT-547 (300 nM and 10 μM) and geldanamycin (300 nM) for 24 h. No increase in HSF-1 or HSP70 was observed following the treatment with NCT-547, whereas geldanamycin highly upregulated HSP70 and increased nuclear accumulation of HSF-1. Intensity of nuclear HSF-1 (green) and cytosolic HSP70 (red) is represented in arbitrary units as defined by the software using the intensity profile tool. Gelda; geldanamycin.

***Supplementary Figure S7***

**
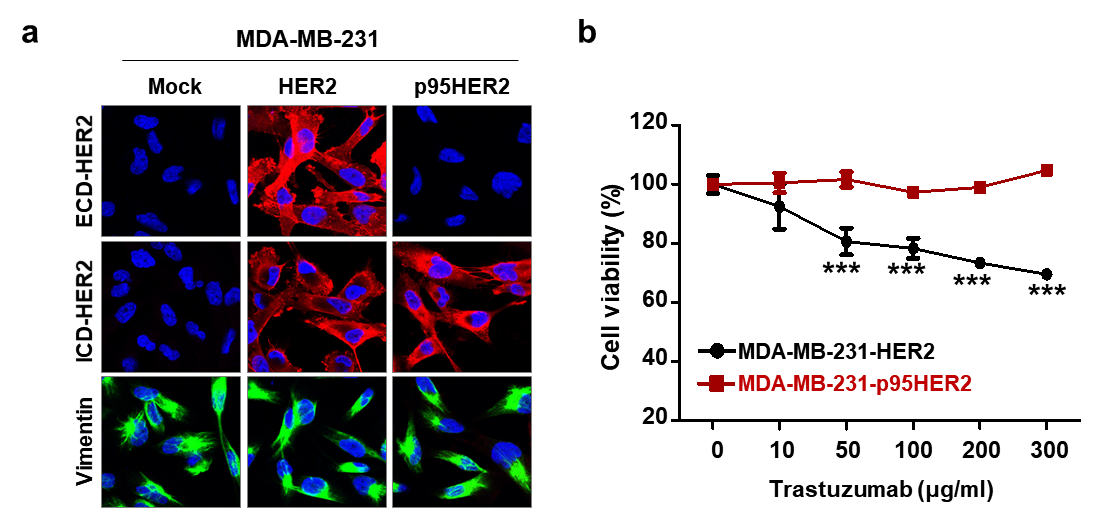
**

**Supplementary Figure S7.** Generation and characterization of HER2- and p95HER2-overexpressing MDA-MB-231 cells.

**a** Immunofluorescence analysis of HER2 and p95HER2. Cells were immunostained with ECD-HER2 or ICD-HER2 (CB11) antibody and counterstained with DAPI (blue). Vimentin (green) was stained to demonstrate cellular features in MDA-MB-231 TNBC cells. **b** Effect of trastuzumab (0-300 μg/ml, 72 h) on viability of HER2-/p95HER2-overexpressing MDA-MB-231 cells as determined by MTS assay (***p<0.001). At least three independent experiments were performed and the data are shown as mean values ± SEM. Data were analyzed by two-way ANOVA followed by Bonferroni’s post hoc test.

***Supplementary Figure S8***


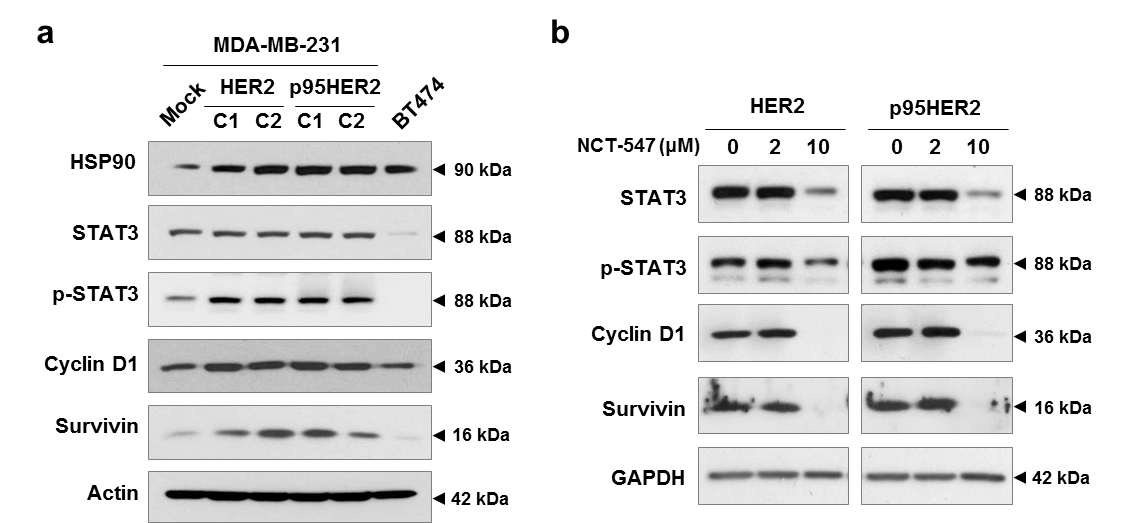


**Supplementary Figure S8.** Influence of NCT-547 on protein content of STAT3 and its downstream factors in HER2- and p95HER2-overexpressing cells.

**a** Immunoblot analyses of HSP90, STAT3, phospho-STAT3 (Tyr705), cyclin D1 and survivin protein expression in parental-, HER2- and p95HER2-overexpressing MDA-MB-231 with two clones (C1 and C2). Actin was used as a loading control. **b** Immunoblot analyses of HSP90, STAT3, phospho-STAT3 (Tyr705), cyclin D1 and survivin protein expression in HER2- and p95HER2-overexpressing MDA-MB-231 cells after NCT-547 (2 µM and 10 µM) challenge. GAPDH was used as a loading control.

***Supplementary Figure S9***

**
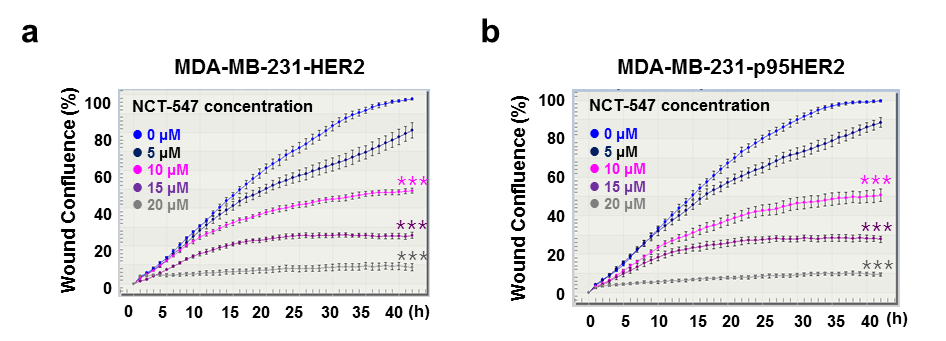
**

**Supplementary Figure S9.** NCT-547 treatment inhibits migratory ability of HER2- and p95HER2-overexpressing cells.

**a-b** Effect of NCT-547 on cell migration of MDA-MB-231-HER2 (**a**) and MDA-MB-231-p95HER2 (**b**) cells. After NCT-547 treatment (0-20 µM), kinetic analysis of cell migration was conducted using an IncuCyte™ Live-Cell Imaging System for the indicated time durations. Kinetic graph of cell migration represents the relative wound density (***p<0.001).

***Supplementary Figure S10***


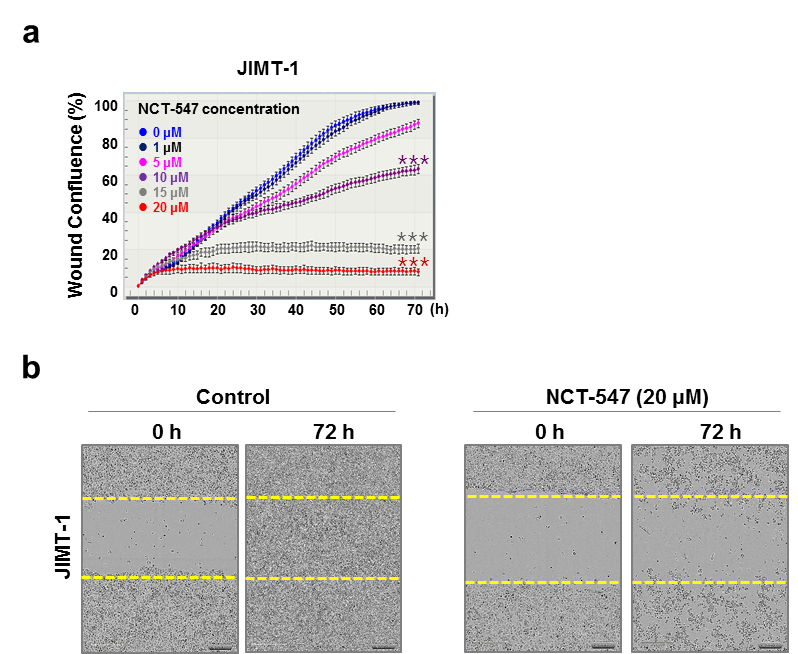


**Supplementary Figure S10.** NCT-547 inhibits migratory ability of trastuzumab-resistant JIMT-1 cells.

**a-b** Effect of NCT-547 on cell migration. **a** Following NCT-547 treatment (0-20 µM), cell migration kinetic analysis was conducted using an IncuCyte™ Live-Cell Imaging System for the indicated time durations. The kinetic graph of cell migration represents the relative wound density (***p<0.001). **b** Representative images show wound closure by cell migration at 0 and 72 h in the presence or absence of 20 µM NCT-547. The yellow lines indicate the initial scratch areas (width, 700-800 µm) and the gray regions represent the empty space not covered by cells.

***Supplementary Figure S11***


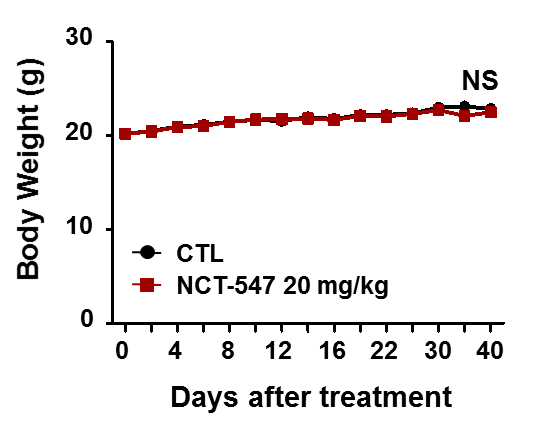


**Supplementary Figure S11.** Changes in body weight of the xenograft mice after exposure to NCT-547 or vehicle control.

JIMT-1 cells (3 × 10^6^) were injected into the mammary fat pads of BALB/c nude mice. Mice were administered intraperitoneally with NCT-547 (20 mg/kg, body weight, every other day) or solvent control for 40 days (n=9/each group). No significant difference was observed in the body weight between the control and NCT-547 treated groups (NS; not significant). Data was analyzed by two-way ANOVA followed by Bonferroni’s post hoc test.

***Supplementary Figure S12***


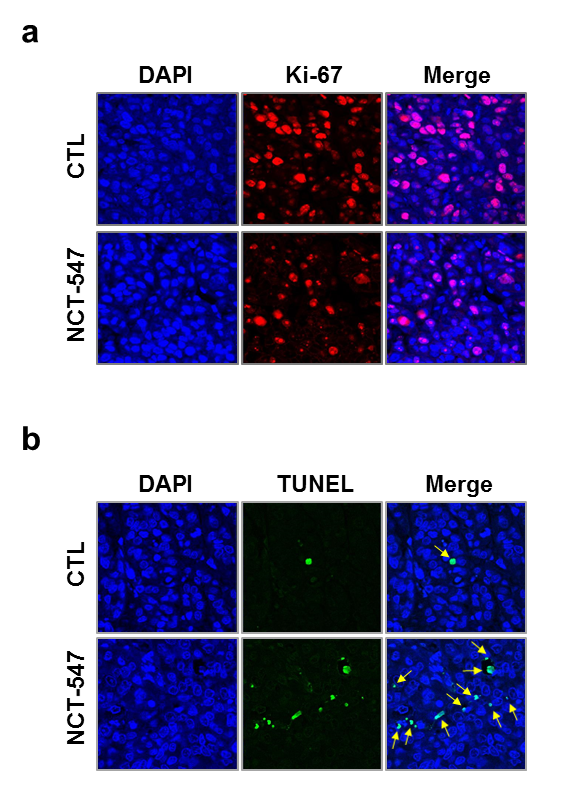


**Supplementary Figure S12.** Influence of NCT-547 on Ki-67 expression and apoptosis *in vivo*

**a** Effect of NCT-547 on proliferating tumor cells was examined by Ki-67 staining. Tissue sections were stained for Ki-67 (red) and nuclei were stained DAPI (blue). **b** NCT-547-induced apoptosis was measured by TUNEL assay and nuclei were counterstained with DAPI (blue). Original magnification: ×500.

***Supplementary Figure S13***


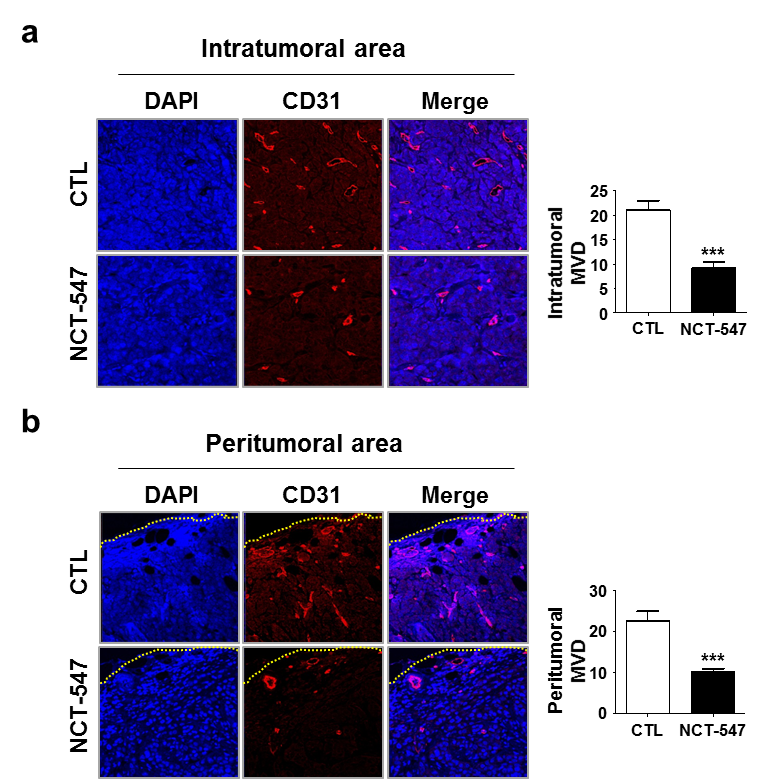


**Supplementary Figure S13.** NCT-547 administration resulted in a significant reduction in tumor angiogenesis.

**a-b** A specific endothelial marker CD31 (red) and DAPI (blue) were used for immunostaining to investigate angiogenesis in tumor tissues after NCT-547 administration, as determined by MVD. The number of CD31-positive microvessels in the intratumoral- and peritumoral areas was quantified (***p<0.001). Fluorescent images of CD31-positive microvessels from intratumoral areas (**a**) and peritumoral areas (**b**) of tumors were taken using confocal microscopy (original magnification: ×200). Yellow dotted lines indicate peritumoral margin.

***Supplementary Figure S14***


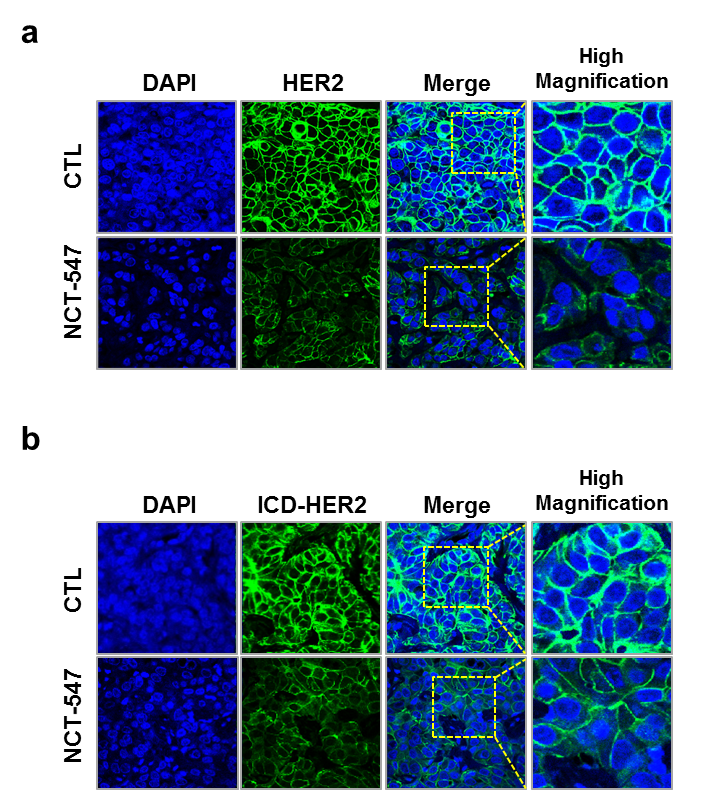


**Supplementary Figure S14.** NCT-547 downregulates intracellular domain (ICD)-HER2 *in vivo.*

**a-b** NCT-547 administration resulted in marked downregulation of HER2 (**a**) and ICD-HER2 (**b**) in vivo, as determined by immunostaining of full-length HER2 and ICD-HER2. All images were taken with a confocal microscope (original magnification: x500). The selected areas in merged images are shown at high magnification (×1000).

***Supplementary Figure S15***


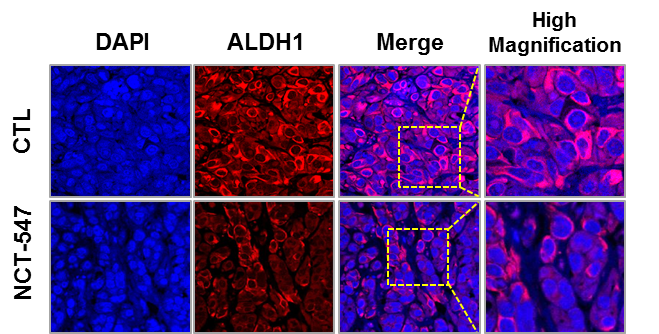


**Supplementary Figure S15.** Influence of NCT-547 on ALDH1 expression *in vivo.*

NCT-547-treated mice showed reduced expression of ALDH1 in vivo. Tumor tissue sections were immunostained for ALDH1A1 (red) and DAPI (blue). Original magnification: ×500. The selected areas in merged images are shown at high magnification (×1000).
